# Disorder in the anionic part of *catena*-poly[[(pyrazine-2-carboxyl­ato)copper(II)]-μ-pyrazine-2-carboxyl­ato]

**DOI:** 10.1107/S1600536812012378

**Published:** 2012-03-31

**Authors:** Joselyn Albanez, Iván Brito, Alejandro Cárdenas, Matías López-Rodríguez

**Affiliations:** a Departamento de Química, Facultad de Ciencias Básicas, Universidad de Antofagasta, Casilla 170, Antofagasta, Chile; b Departamento de Física, Facultad de Ciencias Básicas, Universidad de Antofagasta, Casilla 170, Antofagasta, Chile; c Instituto de Bio-Orgánica ‘Antonio González’, Universidad de La Laguna, Astrofísico Francisco Sánchez No. 2, La Laguna, Tenerife, Spain

## Abstract

The title compound, [Cu(C_5_H_3_N_2_O_2_)_0.88_(C_6_H_4_NO_2_)_1.12_]_*n*_, is characterized by disorder of the anion, resulting from a statistical occupation in a 0.44 (3):0.56 (3) ratio of pyrazine-2-carboxylate and pyridine-2-carboxylate. The compound was isolated during attempts to synthesize a mixed-ligand coordination polymer by solvothermal reaction between copper(II) nitrate and equimolar mixtures of pyrazine-2-carboxylic acid and pyridine-2-carb­oxy­lic acid in a mixture of water and EtOH. The difference in the two components of the compound is due to substitutional disorder of a CH group for one of the N atoms of the pyrazine ring which share the same site in the structure. In the crystal structure, the Cu^II^ atom lies on an inversion centre and is six-coordinated in a distorted N_2_O_4_ geometry. The carboxyl­ate group carbonyl O atoms are weakly coordinated to an equivalent Cu^II^ atom that is translated one unit cell in the *a*-axis direction, thus forming a polymeric chain through carboxyl­ate bridges.

## Related literature

For background to coordination chemistry, see: Blake *et al.* (1999 ▶); Brito *et al.* (2011 ▶). For related compounds with pyridine-2-carboxyl­ate ligands, see: Żurowska *et al.* (2007 ▶). For other similar compounds of the type *M*(C_5_H_3_N_2_O_2_)_2_ where *M* = Cu^II^, Ni^II^, Ag^I^, Co^II^, see: Gao *et al.* (2007 ▶); Jaber *et al.* (1994 ▶); Klein *et al.* (1982 ▶).
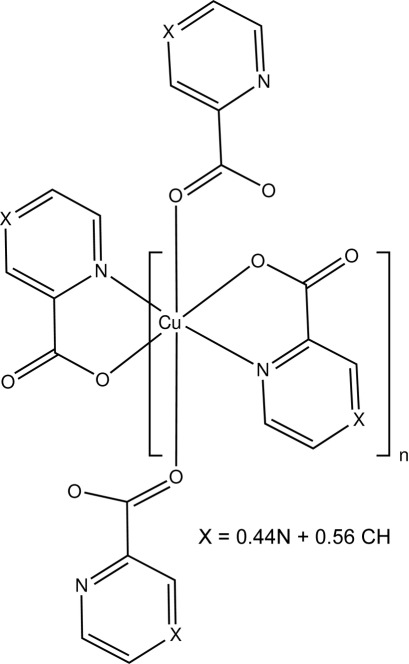


## Experimental

### 

#### Crystal data

[Cu(C_5_H_3_N_2_O_2_)_0.88_(C_6_H_4_NO_2_)_1.12_]
*M* *_r_* = 308.62
Triclinic, 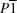

*a* = 5.1912 (10) Å
*b* = 7.3362 (15) Å
*c* = 8.0760 (16) Å
α = 72.38 (3)°
β = 73.35 (3)°
γ = 72.06 (3)°
*V* = 272.47 (9) Å^3^
*Z* = 1
Mo *K*α radiation
μ = 2.02 mm^−1^
*T* = 295 K
0.40 × 0.36 × 0.18 mm

#### Data collection

Nonius KappaCCD area-detector diffractometer
Absorption correction: multi-scan (*MULABS*; Spek, 2009 ▶; Blessing, 1995 ▶) *T*_min_ = 0.459, *T*_max_ = 0.690
2422 measured reflections
1222 independent reflections
1196 reflections with *I* > 2σ(*I*)
*R*_int_ = 0.018

#### Refinement

*R*[*F* ^2^ > 2σ(*F* ^2^)] = 0.022
*wR*(*F* ^2^) = 0.059
*S* = 1.10
1222 reflections
102 parameters
H atoms treated by a mixture of independent and constrained refinement
Δρ_max_ = 0.31 e Å^−3^
Δρ_min_ = −0.28 e Å^−3^

### 

Data collection: *COLLECT* (Nonius, 2000 ▶); cell refinement: *DENZO-SMN* (Otwinowski & Minor, 1997 ▶); data reduction: *DENZO-SMN*; program(s) used to solve structure: *SHELXS97* (Sheldrick, 2008 ▶); program(s) used to refine structure: *SHELXL97* (Sheldrick, 2008 ▶); molecular graphics: *ORTEP-3 for Windows* (Farrugia, 1997 ▶) and *PLATON* (Spek, 2009 ▶); software used to prepare material for publication: *publCIF* (Westrip, 2010 ▶).

## Supplementary Material

Crystal structure: contains datablock(s) I, global. DOI: 10.1107/S1600536812012378/mw2061sup1.cif

Structure factors: contains datablock(s) I. DOI: 10.1107/S1600536812012378/mw2061Isup2.hkl

Additional supplementary materials:  crystallographic information; 3D view; checkCIF report

## Figures and Tables

**Fig. 1.**
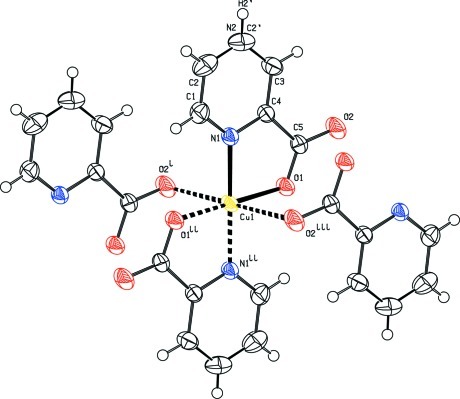
A view of the molecular structure of (I) with the atom-numbering scheme. The 5-position of the aromatic ring is partly N(0.46) and partly a CH group (0.54). Displacement ellipsoids are drawn at the 30% probability level and H atoms are shown as small spheres of arbitrary radii. Symmetry codes i: -1 + x, y, z; ii:1 - x, 1 - y, 1 - z; iii:2 - x, 1 - y, 1 - z.

**Fig. 2.**
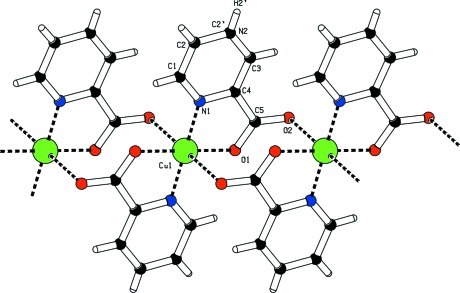
Part of the crystal structure showing the polymeric structure with linear chains extending parallel to [100] direction.

## supplementary crystallographic information

### Comment

The design of polymeric organic-inorganic materials with novel topologies and structural motifs is of current interest in the field of coordination chemistry, (Blake *et al.*, 1999). This paper forms part of our continuing study of the synthesis, structural characterization and physical properties of coordination polymers (Brito *et al.*, 2011). The title compound was isolated during attempts to synthesize a mixed-ligand coordination polymer by solvothermal reaction. Our initial model, with mixed ligands, was not considered because the compound crystallizes in the space group *P*1 with Cu^II^ located at a centre of symmetry so the model was changed to considering both ligands separately . A marginally better refinement is obtained when pyridine-2-carboxylate is used *versus* pyrazine-2-carboxylate as the ligand, (*R1* = 0.024 *versus R1* = 0.025 respectively) although the bond distances are, admittedly, more consistent with pyrazine-2-carboxylate as opposed to pyridine-2-carboxylate ligand. However, we observed that modeling the ligand as pyrazine-2-carboxylate led to displacement parameter for the putative N2 atom being considerably larger than those of the neighboring carbon atoms. Further refinements shown evidence for an H atom of partial occupancy at a distance from N2 atom which suggest this site is partially occupied by a CH group. Consequently, a crystal model containing both bis(pyridinecarboxylate)copper(II) and bis(pyridinecarboxylate)copper(II) species was used and the final value for *R1*is 0.0216 with the displacement parameter for the site occupied by both N2 and C2' is now comparable to those of the adjacent atoms. The title compound, Fig.1, shows only slight variations in molecular geometry and supramolecular organization from the structure described for bis(pyridinecarboxylate)copper(II),Żurowska *et al.* (2007). The Cu^II^ atom is coordinated in a bidentate fashion by two O atoms and two N atoms from symmetry-related pyrazine-2-carboxylate anions. The carboxylate group carbonyl O atoms are weakly coordinated to an equivalent copper atom that is translated one unit cell in the *x* direction, thus forming a polymeric one-dimensional chain through a carboxylate bridge in a slightly distorted octahedral geometry, Fig.2.

### Experimental

The title compound was obtained by the solvothermal reaction of a mixture of copper(II) nitrate (1 mmol), pyrazine-2-carboxylic acid (0.5 mmol), pyridine-2-carboxylic acid (0.5 mmol) in water (5 ml) and EtOH (1 ml) in an acid digestion bomb heated at 150 °C for 3 d and then cooled to room temperature. Suitable single crystals grow upon cooling of the solution to room temperature. Only a few blue single crystals were obtained due to low yield of the reaction and no spectroscopic data were recorded.

### Refinement

Atom H2' was found in difference map and positioned geometrically at a distance of 0.93 Å from the parent C2' atom; a riding model was used during the refinement process. The remaining H atoms were located in a difference Fourier syntheses and were refinend isotropically; C—H range is 0.86 (3)–0.93 (3) Å. The C2' and N2 atoms were refined using the same position and atomic displacement parameters (adp), due to substitutional disorder of a CH group (C2' & H2') for one of the N atoms with SOFs 0.56 (3), 0.44 (3) respectively.

### Crystal data

**Table:**

[Cu(C_5_H_3_N_2_O_2_)_0.88_(C_6_H_4_NO_2_)_1.12_]	*Z* = 1
*M**_r_* = 308.62	*F*(000) = 155
Triclinic, *P*1	*D*_x_ = 1.881 Mg m^−^^3^
Hall symbol: -P 1	Mo *K*α radiation, λ = 0.71073 Å
*a* = 5.1912 (10) Å	Cell parameters from 2320 reflections
*b* = 7.3362 (15) Å	θ = 4.2–27.5°
*c* = 8.0760 (16) Å	µ = 2.02 mm^−^^1^
α = 72.38 (3)°	*T* = 295 K
β = 73.35 (3)°	Block, blue
γ = 72.06 (3)°	0.40 × 0.36 × 0.18 mm
*V* = 272.47 (9) Å^3^	

### Data collection

**Table:**

Nonius KappaCCD area-detector diffractometer	1222 independent reflections
Radiation source: fine-focus sealed tube	1196 reflections with *I* > 2σ(*I*)
Graphite monochromator	*R*_int_ = 0.018
φ and ω scans with κ offsets	θ_max_ = 27.5°, θ_min_ = 4.2°
Absorption correction: multi-scan (*MULABS*; Spek, 2009; Blessing, 1995)	*h* = −6→6
*T*_min_ = 0.459, *T*_max_ = 0.690	*k* = −9→8
2422 measured reflections	*l* = −10→10

### Refinement

**Table:**

Refinement on *F*^2^	Primary atom site location: structure-invariant direct methods
Least-squares matrix: full	Secondary atom site location: difference Fourier map
*R*[*F*^2^ > 2σ(*F*^2^)] = 0.022	Hydrogen site location: inferred from neighbouring sites
*wR*(*F*^2^) = 0.059	H atoms treated by a mixture of independent and constrained refinement
*S* = 1.10	*w* = 1/[σ^2^(*F*_o_^2^) + (0.0201*P*)^2^ + 0.1488*P*] where *P* = (*F*_o_^2^ + 2*F*_c_^2^)/3
1222 reflections	(Δ/σ)_max_ = 0.029
102 parameters	Δρ_max_ = 0.31 e Å^−^^3^
0 restraints	Δρ_min_ = −0.28 e Å^−^^3^

### Special details

**Table:**

Geometry. All s.u.'s (except the s.u. in the dihedral angle between two l.s. planes) are estimated using the full covariance matrix. The cell s.u.'s are taken into account individually in the estimation of s.u.'s in distances, angles and torsion angles; correlations between s.u.'s in cell parameters are only used when they are defined by crystal symmetry. An approximate (isotropic) treatment of cell s.u.'s is used for estimating s.u.'s involving l.s. planes.
Refinement. Refinement of *F*^2^ against ALL reflections. The weighted *R*-factor *wR* and goodness of fit *S* are based on *F*^2^, conventional *R*-factors *R* are based on *F*, with *F* set to zero for negative *F*^2^. The threshold expression of *F*^2^ > 2σ(*F*^2^) is used only for calculating *R*-factors(gt) *etc*. and is not relevant to the choice of reflections for refinement. *R*-factors based on *F*^2^ are statistically about twice as large as those based on *F*, and *R*- factors based on ALL data will be even larger.

### Fractional atomic coordinates and isotropic or equivalent isotropic displacement parameters (Å^2^)

**Table:**

	*x*	*y*	*z*	*U*_iso_*/*U*_eq_	Occ. (<1)
Cu1	0.5000	0.5000	0.5000	0.02958 (12)	
O1	0.2209 (2)	0.6273 (2)	0.35621 (16)	0.0330 (3)	
O2	−0.2197 (3)	0.7996 (2)	0.3757 (2)	0.0447 (4)	
N1	0.2291 (3)	0.6433 (2)	0.67259 (18)	0.0278 (3)	
N2	−0.1986 (4)	0.8650 (3)	0.8789 (3)	0.0477 (6)	0.44 (3)
C2'	−0.1986 (4)	0.8650 (3)	0.8789 (3)	0.0477 (6)	0.56 (3)
H2'	−0.3423	0.9394	0.9485	0.105 (13)*	0.56 (3)
C1	0.2491 (4)	0.6471 (3)	0.8334 (2)	0.0371 (4)	
C2	0.0365 (5)	0.7570 (4)	0.9378 (3)	0.0472 (5)	
C3	−0.2176 (4)	0.8611 (3)	0.7148 (3)	0.0368 (4)	
C4	−0.0027 (3)	0.7483 (2)	0.6142 (2)	0.0272 (3)	
C5	−0.0066 (3)	0.7277 (3)	0.4335 (2)	0.0291 (3)	
H1	0.410 (5)	0.577 (4)	0.867 (3)	0.042 (6)*	
H2	0.053 (6)	0.752 (5)	1.042 (4)	0.069 (9)*	
H3	−0.372 (5)	0.935 (4)	0.668 (3)	0.042 (6)*	

### Atomic displacement parameters (Å^2^)

**Table:**

	*U*^11^	*U*^22^	*U*^33^	*U*^12^	*U*^13^	*U*^23^
Cu1	0.02016 (16)	0.04007 (19)	0.02635 (16)	0.00535 (11)	−0.01042 (11)	−0.01248 (12)
O1	0.0260 (6)	0.0423 (7)	0.0293 (6)	0.0036 (5)	−0.0123 (5)	−0.0124 (5)
O2	0.0309 (7)	0.0543 (9)	0.0473 (8)	0.0089 (6)	−0.0228 (6)	−0.0156 (7)
N1	0.0237 (7)	0.0337 (7)	0.0258 (6)	−0.0033 (6)	−0.0074 (5)	−0.0084 (5)
N2	0.0470 (12)	0.0488 (12)	0.0427 (11)	−0.0045 (9)	0.0029 (8)	−0.0233 (9)
C2'	0.0470 (12)	0.0488 (12)	0.0427 (11)	−0.0045 (9)	0.0029 (8)	−0.0233 (9)
C1	0.0379 (10)	0.0473 (11)	0.0285 (8)	−0.0089 (8)	−0.0116 (7)	−0.0095 (8)
C2	0.0606 (14)	0.0542 (13)	0.0303 (9)	−0.0172 (11)	−0.0035 (9)	−0.0176 (9)
C3	0.0280 (9)	0.0350 (9)	0.0434 (10)	−0.0006 (7)	−0.0051 (8)	−0.0132 (8)
C4	0.0226 (7)	0.0282 (8)	0.0302 (8)	−0.0045 (6)	−0.0066 (6)	−0.0070 (6)
C5	0.0247 (8)	0.0302 (8)	0.0317 (8)	−0.0016 (6)	−0.0115 (6)	−0.0064 (6)

### Geometric parameters (Å, º)

**Table:**

Cu1—O1^i^	1.9476 (13)	N2—C2	1.361 (3)
Cu1—O1	1.9476 (13)	N2—C3	1.366 (3)
Cu1—N1^i^	1.9694 (16)	C1—C2	1.381 (3)
Cu1—N1	1.9694 (16)	C1—H1	0.91 (2)
O1—C5	1.283 (2)	C2—H2	0.86 (3)
O2—C5	1.227 (2)	C3—C4	1.381 (3)
N1—C4	1.341 (2)	C3—H3	0.93 (3)
N1—C1	1.342 (2)	C4—C5	1.519 (2)
			
O1^i^—Cu1—O1	180.0	C2—C1—H1	122.7 (15)
O1^i^—Cu1—N1^i^	83.40 (6)	N2—C2—C1	120.51 (19)
O1—Cu1—N1^i^	96.60 (6)	N2—C2—H2	121 (2)
O1^i^—Cu1—N1	96.60 (6)	C1—C2—H2	119 (2)
O1—Cu1—N1	83.40 (6)	N2—C3—C4	120.01 (18)
N1^i^—Cu1—N1	180.0	N2—C3—H3	121.1 (15)
C5—O1—Cu1	115.04 (10)	C4—C3—H3	118.9 (15)
C4—N1—C1	118.73 (16)	N1—C4—C3	121.49 (16)
C4—N1—Cu1	112.38 (11)	N1—C4—C5	114.22 (14)
C1—N1—Cu1	128.89 (13)	C3—C4—C5	124.29 (16)
C2—N2—C3	118.16 (18)	O2—C5—O1	125.62 (16)
N1—C1—C2	121.11 (19)	O2—C5—C4	119.76 (16)
N1—C1—H1	116.2 (16)	O1—C5—C4	114.58 (14)
			
O1^i^—Cu1—O1—C5	−140 (100)	C2—N2—C3—C4	0.5 (3)
N1^i^—Cu1—O1—C5	175.17 (13)	C1—N1—C4—C3	0.8 (3)
N1—Cu1—O1—C5	−4.83 (13)	Cu1—N1—C4—C3	−179.34 (14)
O1^i^—Cu1—N1—C4	−178.65 (12)	C1—N1—C4—C5	−178.06 (15)
O1—Cu1—N1—C4	1.35 (12)	Cu1—N1—C4—C5	1.77 (18)
N1^i^—Cu1—N1—C4	144 (100)	N2—C3—C4—N1	−1.0 (3)
O1^i^—Cu1—N1—C1	1.16 (17)	N2—C3—C4—C5	177.76 (18)
O1—Cu1—N1—C1	−178.84 (17)	Cu1—O1—C5—O2	−170.82 (16)
N1^i^—Cu1—N1—C1	−37 (100)	Cu1—O1—C5—C4	6.92 (19)
C4—N1—C1—C2	−0.2 (3)	N1—C4—C5—O2	172.07 (17)
Cu1—N1—C1—C2	−179.98 (15)	C3—C4—C5—O2	−6.8 (3)
C3—N2—C2—C1	0.1 (3)	N1—C4—C5—O1	−5.8 (2)
N1—C1—C2—N2	−0.3 (3)	C3—C4—C5—O1	175.33 (17)

Symmetry code: (i) −*x*+1, −*y*+1, −*z*+1.
